# A Busca Continua por Melhores Marcadores que Auxiliem no Diagnóstico e na Prevenção da Fibrilação Atrial e suas Complicações

**DOI:** 10.36660/abc.20250044

**Published:** 2025-03-12

**Authors:** Dario Celestino Sobral

**Affiliations:** 1 Universidade de Pernambuco Faculdade de Ciências Médicas Recife PE Brasil Faculdade de Ciências Médicas - Universidade de Pernambuco, Recife, PE – Brasil; 2 PROCAPE Hospital do Coração Prof. Luiz Tavares Recife PE Brasil PROCAPE Hospital do Coração Prof. Luiz Tavares, Recife, PE – Brasil

**Keywords:** Fibrilação Atrial Assintomática, Rastreamento de Fibrilação Atrial, Acidente Vascular Cerebral Embólico sem Causa Definida

Embora a fibrilação atrial (FA) seja a arritmia cardíaca mais estudada com milhares de publicações em revistas indexadas na última década, várias questões permanecem carentes de resposta, desde a sua etiopatogenia, o seu diagnóstico e tratamento e principalmente sobre como conduzir os pacientes no sentido de prevenir recidivas dessa arritmia e suas complicações.

Identificar os pacientes de maior suscetibilidade para a FA buscando marcadores de risco confiáveis tem sido o objetivo de diversas pesquisas, a maior dificuldade é consequência de ser uma arritmia de caráter multifatorial. Desde mutações genéticas, processos inflamatórios, presença de fibrose atrial e até mesmo condições socioambientais podem estar relacionadas a sua etiologia.^1^

Como bem destacado no artigo de Elbarbary et al.^2^ nesta edição dos Arquivos Brasileiros de Cardiologia, a fibrilação atrial paroxística (FAP) tem alta incidência nessa forma de apresentação e a monitorização eletrocardiográfica prolongada, sobretudo, usar o monitor *loop* implantável pode revelar a presença de FAP em cerca de um terço dos pacientes atendidos com acidente vascular cerebral embólico de causa indeterminada (AVCECI) que estavam em ritmo sinusal na ocasião do atendimento.

O estudo publicado recentemente de autoria de Vinter et al.^3^ desenvolvido na Dinamarca revelou que as complicações mais frequentes a longo prazo de pacientes atendidos em hospital com FA foram a insuficiência cardíaca em primeiro lugar, seguida de acidente vascular cerebral e mostrou também que apesar dos avanços na prevenção primária dessas complicações os resultados não são satisfatórios como era esperado. Tais evidências destacam a importância da pesquisa conduzida por Elbarbary et al.^2^ buscando a identificação de parâmetros clínicos e laboratoriais capazes de prever o risco de FAP e assim melhorar a eficiência de medidas preventivas.

O objetivo da pesquisa de Elbarbary et al.^2^ foi avaliar se a combinação de diferentes variáveis poderia apresentar vantagens na previsão da ocorrência de FAP em pacientes com AVCECI. Além de adotar os parâmetros que definem a presença de cardiopatia atrial, os autores avaliaram outras medidas eletrocardiográficas e o registro do Holter por 7 dias durante internação hospitalar. Outro objetivo do trabalho era obter com os resultados, o aumento da sensibilidade decorrente da aplicação dos métodos empregados podendo, em muitos casos, evitar a indicação do implante do dispositivo para registro eletrocardiográfico por longos períodos (*loop recorder*) que tem custo financeiro elevado, inacessível para populações de países de baixa renda.

Esses autores constataram nos pacientes com AVCECI a prevalência de cardiopatia atrial com os fatores hipertensão arterial e a relação E/e- >12 no ecocardiograma como preditores independentes para esta condição e a análise de regressão multivariada identificou os parâmetros Index da Velocidade da Força Terminal da onda P em V1 no eletrocardiograma (ECG) >5000 Mv.ms, o índice de volume atrial esquerdo >34ml/m^2^ e a fração de ejeção ventricular esquerda <45% medidos no ecocardiograma como preditores de FA de recente começo.

## Podemos aplicar os resultados de Elbarbary na prática clínica?

Para responder a essa questão, são necessárias algumas considerações: Por se tratar de uma doença com fisiopatologia complexa com diversos fatores envolvidos no seu surgimento, dificilmente encontraremos biomarcadores que sejam úteis para definir os riscos cardiovasculares de todos os pacientes. Muito embora muitas pesquisas empregando número adequado de pacientes e com metodologia apropriada tenham apresentados resultados promissores para alguns biomarcadores num determinado grupo de pacientes, mas não para uma maioria considerável de pacientes.^4^ Nesse contexto, o marcador mais abrangente é sem dúvida a idade, entretanto, este é um marcador que quase sempre está associado a comorbidades que às vezes sobressaem na avaliação de risco. Além disso, a deterioração biológica que acompanha o processo de envelhecimento não é uniforme entre os indivíduos nem mesmo entre os diferentes órgãos.^5^

Um estudo mostrou que a diferença na idade verificada no ECG com recursos de Inteligência Artificial (ECG-IA) e a idade cronológica, esteve relacionada com risco de morte associada com infarto agudo do miocárdio, insuficiência cardíaca e FA.^6,7^ Esses achados destacam o potencial do ECG-AI como novo biomarcador custo-efetivo para o envelhecimento cardiovascular e para a estratificação de risco. Entretanto, a sua incorporação à prática clínica depende de novos estudos que preencham algumas lacunas apontadas pela pesquisa, como por exemplo, a reprodutibilidade dos achados no mesmo indivíduo ao longo do tempo e qual é o papel de fatores genéticos e socioambientais, além dos tradicionais fatores de risco.^5,6^

Também merecem a nossa atenção as pesquisas abordando os chamados "fatores de risco não-tradicionais" para FA, trazendo novas evidências sobre a importância do estilo de vida e os distúrbios do sono onde a ativação do nervoso simpático parece ser o mecanismo principal.^8^ O reconhecimento da FA como causa de AVCECI implica numa cuidadosa abordagem voltada para a prevenção secundária já que é bem conhecido o alto risco de recorrência de eventos embólicos cerebrais. Apesar disso, o manuseio desses pacientes no mundo real carece de evidências consistentes. Estudo de Sánchez-Sáez et al.^9^ mostrou que o tratamento inicialmente prescrito após o evento isquêmico cerebral nem sempre segue as recomendações das Diretrizes. Além do mais, a adesão e manutenção do tratamento por parte dos médicos e dos pacientes é fundamental para evitar recorrência de eventos tromboembólicos cerebrais.^10^

Em conclusão, o artigo de Elbarbary et al.^2^ com suas limitações, reconhecidas pelos próprios autores, fornece conhecimentos que despertam a necessidade de pesquisas adicionais na busca de informações consistentes que ajudem a prevenir o AVCECI secundário à FA antes da primeira ocorrência. A evolução das pesquisas avança indicando que a abordagem dos pacientes deve ser sempre individualizada com a utilização de recursos principalmente derivados da genética e da inteligência artificial e que em futuro próximo poderão ser utilizados em benefício dos pacientes.
