# Supplementary material for: A Busca Continua por Melhores Marcadores que Auxiliem no Diagnóstico e na Prevenção da Fibrilação Atrial e suas Complicações
Source: Arq Bras Cardiol. 2025 Mar 12;122(1):e20250044. [Article in Portuguese] doi: 10.36660/abc.20250044 (PMC12013739; doi:10.36660/abc.20250044)
Supplement: Translation

# Translation: The Search Continues for Better Markers That Aid in the Diagnosis and Prevention of Atrial Fibrillation and its Complications

**DOI:** 10.36660/abc.20250044i

**Authors:** Dario Celestino Sobral

**Affiliations:** 1 Recife PE Brazil Faculdade de Ciências Médicas - Universidade de Pernambuco, Recife, PE – Brazil; 2 Recife PE Brazil PROCAPE Hospital do Coração Prof. Luiz Tavares, Recife, PE – Brazil

**Keywords:** Markers of Atrial Fibrillation, Asymptomatic Atrial Fibrillation, Embolic Stroke of Undetermined Cause

Although atrial fibrillation (AF) is the most studied cardiac arrhythmia, with thousands of publications in indexed journals over the last decade, several questions remain unanswered, ranging from its etiopathogenesis, diagnosis, and treatment, to how best to manage patients in order to prevent recurrences of this arrhythmia and its complications.

Identifying patients at higher risk for AF by searching for reliable risk markers has been the goal of several studies, with the greatest difficulty arising from the multifactorial nature of the arrhythmia. Genetic mutations, inflammatory processes, the presence of atrial fibrosis, and even socioenvironmental conditions may be related to its etiology.^1^

As highlighted in the article by Elbarbary et al.^2^ in this edition of the *Arquivos Brasileiros de Cardiologia*, paroxysmal atrial fibrillation (PAF) has a high incidence in this form of presentation, and prolonged electrocardiographic monitoring, particularly using implantable loop monitors, can reveal the presence of PAF in about one-third of patients with an embolic stroke of undetermined source (ESUS) who were in sinus rhythm at the time of presentation.

A recent study by Vinter et al.,^3^ conducted in Denmark, revealed that the most common long-term complications for patients hospitalized with AF were heart failure, followed by ischemic stroke. The study also showed that, despite advances in the primary prevention of these complications, the results have not been as satisfactory as expected. Such evidence highlights the importance of the research conducted by Elbarbary et al.,^2^ which aims to identify clinical and laboratory parameters capable of predicting the risk of PAF and thus improving the effectiveness of preventive measures.

The goal of Elbarbary et al.^2^ research was to assess whether combining different variables could provide advantages in predicting the occurrence of PAF in patients with ESUS. In addition to using parameters that define the presence of atrial heart disease, the authors evaluated other electrocardiographic measures and 7-day Holter monitoring during hospitalization. Another goal was to increase sensitivity through the application of these methods, potentially avoiding the need for the implantation of an electrocardiographic recording device (loop recorder), which is costly and inaccessible for low-income populations.

The authors found that, in patients with ESUS, the prevalence of atrial cardiopathy, characterized by hypertension and E/e- >12 on echocardiogram, were independent predictors for this condition. Multivariate regression analysis identified the P-wave terminal force velocity in lead V1 on electrocardiogram (ECG) greater than 500 µv.ms, left atrial volume index >34 ml/m^2^, and left ventricular ejection fraction <45% as predictors of recent onset AF.

## Can the results from Elbarbary be applied in clinical practice?

To answer this question, several considerations are necessary: since AF is a disease with a complex pathophysiology involving multiple factors, it is unlikely that we will find any biomarkers useful for defining cardiovascular risks in all patients. Although many studies using an adequate number of patients and proper methodology have shown promising results for certain biomarkers in specific patient groups, these findings may not be applicable to a large number of patients.^4^ In this context, the most comprehensive marker is undoubtedly age; however, age is almost always associated with comorbidities that sometimes predominate in risk assessments. Furthermore, the biological deterioration that accompanies the aging process is not uniform across individuals or even among different organs.^5^

One study showed that the difference in age as determined by ECG using Artificial Intelligence (AI-ECG) versus chronological age was associated with a risk of mortality related to acute myocardial infarction, heart failure, and AF.^6,7^ These findings highlight the potential of AI-ECG as a new cost-effective biomarker for cardiovascular aging and risk stratification. However, its incorporation into present clinical practice depends on further studies that address gaps identified in the research, such as the reproducibility of findings in the same individual over time and the role of genetic and socioenvironmental factors, in addition to traditional risk factors.^5,6^

Research focusing on so-called "non-traditional risk factors" for AF also deserves attention, providing new evidence on the importance of lifestyle and sleep disorders, in which sympathetic nervous system activation appears to be the main mechanism.^8^

Recognizing AF as the cause of ESUS requires careful management aimed at secondary prevention, given the well-known high risk of recurrent embolic brain events. However, real-world management of these patients shows a dearth of consistent evidence. A study by Sánchez-Sáez et al.^9^ revealed that the initial treatment prescribed after an ischemic stroke does not always follow guideline recommendations. Furthermore, adherence to and maintenance of treatment by both doctors and patients is crucial to prevent the recurrence of thromboembolic brain events.^10^

In conclusion, the article by Elbarbary et al.,^2^ with its limitations acknowledged by the authors themselves, provides insights that highlight the need for further research in the search for consistent information to help prevent ESUS due to AF before its first occurrence. The evolution of research is progressing, indicating that the approach to patients should always be individualized, utilizing resources primarily derived from genetics and artificial intelligence, which may soon be used for the benefit of patients.
